# Prediction of survival after neoadjuvant therapy in locally advanced rectal cancer – a retrospective analysis

**DOI:** 10.3389/fonc.2024.1374592

**Published:** 2024-05-16

**Authors:** Gudrun Piringer, Florian Ponholzer, Josef Thaler, Thomas Bachleitner-Hofmann, Holger Rumpold, Alexander de Vries, Lukas Weiss, Richard Greil, Michael Gnant, Dietmar Öfner

**Affiliations:** ^1^ Department of Hematology and Oncology, Kepler University Hospital, Linz, Austria; ^2^ Department of Internal Medicine IV, Wels-Grieskirchen Medical Hospital, Wels, Austria; ^3^ Medical Faculty, Johannes Kepler University Linz, Linz, Austria; ^4^ Department of Visceral, Transplant and Thoracic Surgery, Center of Operative Medicine, Medical University of Innsbruck, Innsbruck, Austria; ^5^ Division of General Surgery, Department of Surgery, Medical University of Vienna, Vienna, Austria; ^6^ Department of Hematology and Oncology, Ordensklinikum Linz, Linz, Austria; ^7^ Department of Radiotherapy and Radio-Oncology, Feldkirch Hospital, Feldkirch, Austria; ^8^ 3^rd^ Medical Department of Internal Medicine III, Paracelsus Medical University, Salzburg, Austria; ^9^ Salzburg Cancer Research Institute - Center for Clinical Cancer and Immunology Trials, Salzburg, Austria; ^10^ Comprehensive Cancer Center, Medical University, Vienna, Austria

**Keywords:** neoadjuvant radiochemotherapy, prediction of survival, T-downstaging, Ndownstaging, locally advanced rectal cancer

## Abstract

**Purpose:**

The aim of this retrospective analysis was to determine if the response to preoperative radio(chemo)therapy is predictive for survival among patients with locally advanced rectal cancer and may act as a potential surrogate endpoint for disease free survival and overall survival.

**Results:**

Eight hundred seventy-eight patients from five centers were analyzed. There were 304 women and 574 men; the median age was 64.7 years. 77.6% and 22.4% of patients received neoadjuvant radiochemotherapy or short-course radiotherapy, resulting in a pathological complete response in 7.3%. T-downstaging and N-downstaging occurred in 50.5% and 37% of patients after neoadjuvant therapy. In patients with T-downstaging, the 10-year DFS and 10-year OS were 64.8% and 66.8% compared to 37.1% and 45.9% in patients without T-downstaging. N-downstaging resulted in 10-year DFS and 10-year OS in 56.2% and 62.5% compared to 47.3% and 52.3% without N-downstaging. Based on routinely evaluated clinical parameters, an absolute risk prediction calculator was generated for 5-year disease-free survival, and 5-year overall survival.

**Conclusion:**

T-downstaging and N-downstaging after neoadjuvant radiochemotherapy or short-course radiotherapy resulted in better DFS and OS compared to patients without response. Based on clinical parameters, 5-year DFS, and 5-year OS can be predicted using a prediction calculator.

## Introduction

Since the publication of the German trial by Sauer et al. (1), neoadjuvant fluoropyrimidine-based radiotherapy (RCT) followed by total mesorectal excision (TME) became a standard treatment for locally advanced rectal cancers (LARC). The administration of adjuvant chemotherapy is a highly debated issue as several trials and meta-analysis found no or only a marginal survival benefit (2–7). Nevertheless, the consensus-based guidelines from the National Comprehensive Cancer Network and the ESMO guidelines consider adjuvant chemotherapy and the decision should be risk-balanced (8, 9). Despite optimized local treatment with less than 10% recurrence rates, neoadjuvant RCT has not improved overall survival (OS), and distant metastases still occur in 25–30% (1, 10–13). The primary goals of neoadjuvant treatment for LARC are improvement of local tumor control, tumor downstaging and enabling sphincter-sparing surgery. Patients achieving a complete pathological response, i.e. ypT0N0 (pCR), after neoadjuvant RCT have better long-term outcomes than patients without pCR, which was shown in a pooled analysis by Maas et al. (14). Further developments of neoadjuvant therapy aim to improve primary tumor response and patient outcome. Identifying short-time surrogate endpoints for predicting disease-free survival (DFS) and OS are helpful for individualizing adjuvant therapy and follow-up. There have been several publications concerning short-term surrogate for DFS and OS after neoadjuvant RCT or RT such as tumor regression grade (TRG) (15–18) and Neoadjuvant Rectal Score (NAR score) (19). The NAR score (19) was developed as a short-term clinical trial surrogate endpoint to take variables associated with treatment effects beyond pCR into consideration yet simple enough to support a diversity of clinical trial designs. The NAR score is calculated based on data supported by the Valentini nomogram (20) for OS, but only using the clinical T-stage and pathologic T- and N-stages. Of the eight variables used in the Valentini nomogram, only pN and pT are potentially influenced by neoadjuvant therapy. After establishing the NAR score calculation, it was validated using the NSABP R04 trial patient dataset (21, 22). NAR scores in the NSAPB R-04 trial dataset were categorized as low (NAR<8), intermediate (NAR 8–16), and high (NAR >16) based on the tertiles of the observed scores. These categories were significantly associated with OS (p<0.0001) with 5-year OS values of 92%, 89%, and 68%, respectively. TGR is also predictive of therapeutic response in rectal cancer after RCT followed by curative resection. However, various TGR systems have been suggested, with subjective categorization, resulting in interobserver variability (15–18). Furthermore, even regional lymph node status after RCT is an important prognostic factor, the TRG systems only evaluate the primary tumor with no consideration of regional lymph node status. Due to the subjective classification there is a low concordance rate even in experienced gastrointestinal pathologics using the same TRG system. The Mandard (15) and Dworak (16) TRG systems are classified according to five-point grades based on residual tumor and fibrosis, whereas the Ryan TGR system (17), with three-point grading, is a type of modified Mandard TRG system. The 2010 American Joint Committee on Cancer (AJCC) TRG system (18) is a modification of the Ryan TRG system based on the volume of residual primary tumor cells.

In the ABCSG R02 trial we previously demonstrated that downstaging of the N-level and particularly the achievement of lymph node negativity after neoadjuvant RCT with capecitabine and oxaliplatin (ABCSG R02-Study) exerts a statistically significant influence on 5-year OS and DFS in this patient population (23), whereas downstaging at the T-level showed no statistically significant influence on OS and only a borderline significance in DFS. The finding obtained from the ABCSG R02-trial that downstaging of the N-level has a more significant impact on DFS and OS than T-downstaging was now evaluated in a large cohort receiving neoadjuvant RCT or short-time RT. Furthermore, a prediction calculator for 5-year DFS and 5-year OS was developed based on the available clinical data.

## Methods

### Patients

A total of 993 patients with locally advanced and histologically confirmed adenocarcinoma of the rectum with the indication for neoadjuvant RCT or short-term RT were included in this retrospective analysis from six centers in Austria. Patients were included from 2000–2014, and the postoperative follow-up was recorded until March 2019. Patients received a standardized magnetic resonance imaging (MRI) of the pelvis for local staging of the rectal tumor according to an Austrian MRI-Standard Operating Procedure (SOP) to achieve comparable MRI results. The study was approved by the ethics committee in Upper Austria (EK-No: 1074/2018) and was conducted by the Austrian breast and colorectal cancer study group (ABCSG).

### Treatment

All evaluated patients received neoadjuvant RCT concurrent with fluoropyrimidine +/- oxaliplatin or short-RT, followed by surgical resection. Adjuvant chemotherapy was administered according to regional local standards. Surgical techniques included open and laparoscopic approaches.

### Follow-up

During a median follow-up of 69 months, patients were clinically evaluated (history and examination) and were referred to radiological assessment (chest X-ray, abdominal-pelvic CT scan, colonoscopy, and other investigations) as per clinical indication and local standards. DFS was defined as the time between surgery and the first recurrence of rectal cancer (local or distant) or death. OS was defined as the time between surgery and death.

### Statistical analysis

Statistical analysis was performed using IBM SPSS Statistics 26 (IBM Corporation, Armon, NY, USA) and the R software environment (24). In R the package ‘survminer’ was used to plot survival curves (25). The likelihood-ratio chi-squared test was used to identify correlations between categorical variables. Homogeneity of variance was assessed by Levene’s test. Depending on homogeneity of variance, analysis of variance (ANOVA) or Welch-ANOVA was used for comparing continuous variables between groups. For *post-hoc* testing, Tukey’s test and Games-Howell test were used.

Cox’s proportional hazards regression analysis was used for calculating and establishing an absolute risk prediction model for 5-year DFS and 5-year OS based on Jia et al., who provide a detailed explanation of the used model (26). The formula for the absolute risk prediction model was: $R(t)=1-{\left[Su_{0}(t)\right]}^{\text{exp}(\sum_{r=1\;}^{l}{\text{X}}_{r}{\text{β}}_{r}-\;\sum_{r=1\;}^{l}{\bar{\text{X}}}_{r}{\text{β}}_{r})}$ , where R is the risk of the event occurring in the calculated time period t (60 months in this case), l is the number of risk factors, Su_0_ the base survival probability at mean values, X_r_ is the value of the corresponding risk factor, β_r_ the corresponding regression coefficient. This prediction model study is a Type 1a study with a direct model evaluation using the same data as for model development (27).

The log-rank test was used to compare Kaplan-Meier curves. The missing indicator method was used for missing categorical data, except for the regression analysis, as this would lead to inefficient regression coefficients (28). Statistical significance was assumed for a p-value<0.05. Downstaging is defined as the migration to lower T- or N-classification following neoadjuvant therapy. The extent of regional nodal involvement includes the mesorectal and internal iliac nodes based on size and defined morphologic criteria.

## Results

**Figure 1:**
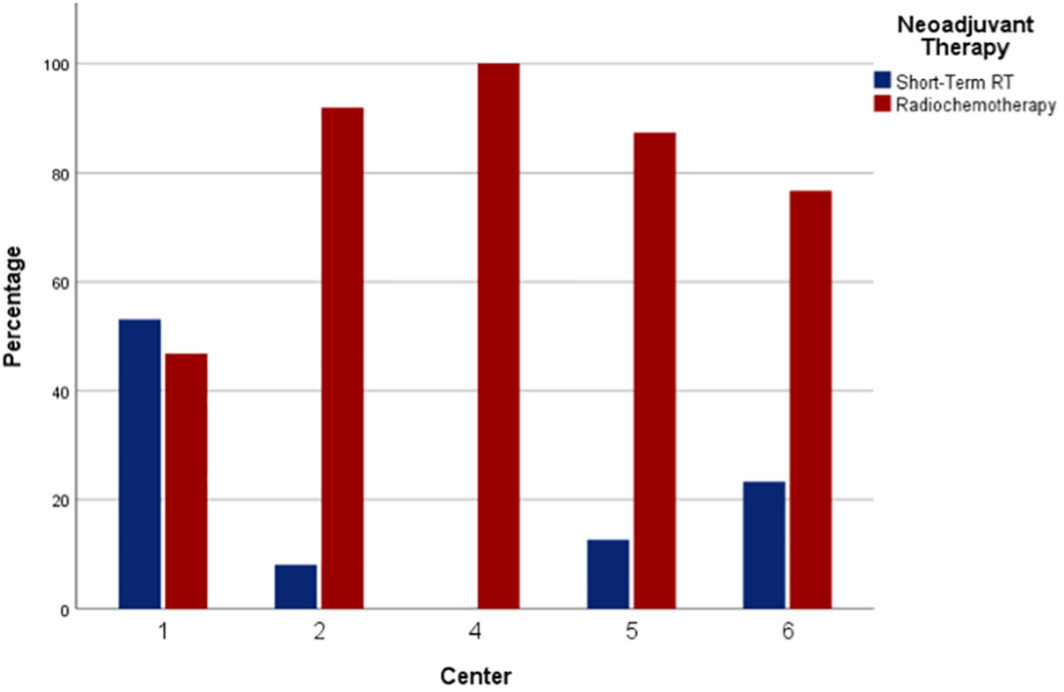
Type of neoadjuvant therapy according to centers.

Nine hundred ninety-three patients were included in this retrospective analysis. One center was excluded due to insufficient follow-up and survival data documentation because most patients had follow-up outside the hospital. After data cleaning 878 patients from five centers were involved in this retrospective analysis with a median age of 64.7 years (min.-max.: 27.5–90.1, range: 62.6), 34.6% (304 patients) were female, and 65.4% (574 patients) were male. The pretreatment stage distribution included cT2, cT3, and cT4 in 3.6%, 86.8%, and 9.6% of patients, and cN0, cN1–2, and nodal status missing in 31.1%, 60.0%, and 8.9%. Due to the primary clinical tumor stage, all patients had an indication for neoadjuvant RCT or short-course RT according to the local standard. Neoadjuvant RCT was done in 77.6%, and short-course RT in 22.4%. Patients in the neoadjuvant RCT had a significantly higher rate of pCR with 8.9% in comparison to 2.1% (p<0.001) in the short-course RT cohort. The pretreatment clinical tumor stage and the performed neoadjuvant therapy modality were not balanced between the five involved centers, as is visualized in **Figure 1** and **Table 1**. Approximately 43.7 days (min.-max.: 0–991, range: 991) after completion of RCT and 16.2 days (min.-max.: 1–161, range: 160) after RT, surgery was performed (p<0.001). Adjuvant chemotherapy was administered to 52.5% of patients (ranging from 39.5% – 81.6% between the five centers). Median follow-up was 78.8 months (min.-max.: 0–256, range: 256). All patients received pathological work-up of the resected specimen, with 7.7% being graded as ypT0, 6.8% as ypT1, 30.5% as ypT2, 49.4% as ypT3 and 4.3% as ypT4. About two-thirds of patients were nodal negative after neoadjuvant therapy, and ypN1 and ypN2 status were found in 21.2% and 11.7%. A pCR (ypT0N0) after neoadjuvant RCT/short-term RT was documented in 7.3% of patients. T-downstaging and N-downstaging occurred in 50.5% and 37% of patients after neoadjuvant therapy. Correspondingly, the UICC tumor stage was down-staged in 47% of patients after neoadjuvant therapy. T-downstaging, N-downstaging, and UICC-downstaging were not significantly better if surgery was done >7 weeks after RCT/RT. After a median follow-up of 55 months, 7.6% of patients (n=67) experienced local recurrence, and 21.2% of patients (n=186) developed distant metastases. The 3-year and 5-year DFS were 73.0 months and 64.3 months, respectively. The 5-year and 10-year OS were 75.8 and 55.8 months, respectively.

**Table 1:** Demographic description of patients.

	Center 1	Center 2	Center 3	Center 4	Center 5	Statistics with p-value	Total
**N (RCT; %)**	256 (120; 46.9)	99 (91; 91.9)	190 (190, 100)	230 (201; 87.4)	103 (79; 76.7)	G ^2^ = 239.28 DF=4<.001	878 (681; 77.6)
**Age (SD; 95% CI)**	64.6 (10.79; 63.30–65.95)	65.6 (11.00; 63.43–67.82)	63.9 (11.53; 62.24–65.54)	64.6 (11.69; 63.11–66.15)	65.6 (11.56; 63.33–67.85)	n.s.	64.7 (11.30; 63.95–65.44)
**Sex (f; %)**	93 (36.3)	36 (36.4)	68 (35.8)	72 (31.3)	35 (34.0)	G^2^ = 1.73 DF=4 n.s.	304 (34.6)
cT-status (%)							
cT2	11 (4.3)	8 (8.1)	0 (0)	10 (4.3)	3 (2.9)	G^2^ = 30.72 DF=8<.001	32 (3.6)
cT3	223 (87.1)	73 (73.7)	169 (88.9)	203 (88.3)	94 (91.3)		762 (86.8)
cT4	22 (8.6)	18 (18.2)	21 (11.1)	17 (7.4)	6 (5.8)		84 (9.6)
cN-status (%)							
negative	63 (24.6)	34 (34.3)	51 (26.8)	99 (43.0)	26 (25.2)	G^2^ = 150.00 DF=8<.001	273 (31.1)
positive	182 (71.1)	59 (59.6)	81 (42.6)	131 (57.0)	74 (71.8)		527 (60.0)
missing or not identifiable	11 (4.3)	6 (6.1)	58 (30.5)	0 (0)	3 (2.9)		78 (8.9)
Neoadjuvant therapy (%)							
RCT	120 (46.9)	91 (91.9)	190 (100)	201 (87.4)	79 (76.7)	G^2^ = 239.28 DF=4<.001	681 (77.6)
RT	136 (53.1)	8 (8.1)	0 (0)	29 (12.6)	24 (23.3)		197 (22.4)
Interval* **last R(C)Tx-OP in days (SD; 95% CI)**	43.8 (78.06; 34.16–53.38)	49.4 (38.15; 41.83–57.04)	31.0 (23.22; 27.5–34.44)	30.2 (48.07; 23.91–36.40)	39.2 (59.94; 26.87–51.43)	F=6.27 df1 = 4 df2 = 320.31<.001	37.6 (56.24; 33.85–41.41)
for RCT	75.3(101.98; 56.82–93.69)	53.2 (37.54; 45.34–60.97)	31.0 (23.22; 27.51–34.44)	32.0 (49.79; 25.10–38.95)	42.9 (64.78; 28.28–57.49)	F=11.66 df1 = 4 df2 = 244.58<.001	43.7 (60.88; 39.08–48.35)
for >=7w RCT	97.4 (123.94; 68.86–125.89)	67.6 (48.10; 53.30–81.87)	71.6 (49.66; 47.70–95.57)	136.8 (187.25; 11.02–262.61	132.0 (206.83; -59.29–323.29)	F=1.23 df1 = 4 df2 = 25.89 n.s.	89.9 (111.81; 72.32–107.45)
Surgery (%)							
AR	6 (2.3)	2 (2.0)	12 (6.3)	0 (0)	9 (8.7)	G^2^ = 86.64 DF=16<.001	29 (3.3)
LAR	197 (77.0)	56 (56.6)	118 (62.1)	180 (78.3)	56 (54.4)		607 (69.1)
Hartmann	0 (0)	0 (0)	9 (4.7)	0 (0)	0 (0)		9 (1.0)
APE	53 (20.7)	40 (40.4)	51 (26.8)	50 (21.7)	38 (36.9)		232 (26.4)
transrectal	0 (0)	1 (1.0)	0 (0)	0 (0)	0 (0)		1 (0.1)

**Figure 2:**
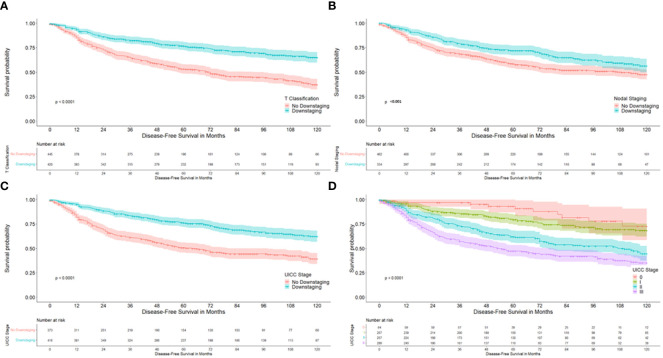
Kaplan-Meier curves with regard to disease free survival. **(A)** according to downstaging at the T-level; **(B)** according to downstaging at the nodal status; **(C)** according to downstaging at UICC stages; **(D)** according to UICC stage.

**Figure 3:**
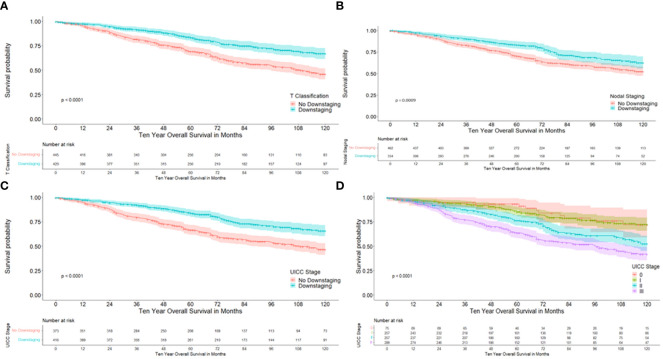
Kaplan-Meier curves with regard to overall survival. **(A)** according to downstaging at the T-level; **(B)** according to downstaging at the nodal status; **(C)** according to downstaging at UICC stages; **(D)** according to UICC stage.

Univariate analysis demonstrated a significantly better 10-year DFS in patients with downstaging in T-level (64.8% versus 37.1%; p<0.001), downstaging in N-level (56.2% versus 47.3%, p 0.001), downstaging in UICC stage (62.1% versus 39.1%), p< 0.001) and with lower UICC stages, as is described in **Table 2** and as can be seen in **Figure 2**. 10-year DFS was 67.3% versus 48.6% in ypT0 versus ypT1–4 (p<0.001) and 72.6% versus 48.3% (p<0.001) in patients with a complete pathological response (ypT0N0) versus no complete response. Furthermore, a significantly better 10-year OS was demonstrated in patients with downstaging in T-level (66.8% versus 45.9%, p<0.001), downstaging in N-level (62.5% versus 52.3%, p 0.001), downstaging in UICC stage (65.6% vs. 46.7%, p<0.001). Lower UICC stages, as shown in **Table 3** and as can be seen in **Figure 3**. 10-year OS was 66.8% versus 54.7% in ypT0 versus ypT1–4 (p 0.022) and 72.1% versus 54.4% (p 0.006) in patients with a complete pathological response (ypT0N0) versus no complete response. Four patients were ypT0 but ypN1.

**Table 2:** 10-year Disease-Free Survival Univariate Analysis.

Factor	10-Year Overall Survival (%)	p-Value
Age Split according to Median <65.8 ≥65.8	62.1 38.1	<.001
Sex Female Male	57.8 46.4	.005
cT cT2 cT3 cT4	53.7 52.5 29.2	.002
cN cN0 cN1 cN2	54.6 53.8 37.9	<.001
Clinical Nodal Status Negative Positive	54.6 49.1	.138
Downstaging @T-level Downstaging No Downstaging	64.8 37.1	<.001
Downstaging @N-level Downstaging No Downstaging	56.2 47.3	.001
Downstaging UICC-level Downstaging No Downstaging	62.1 39.1	<.001
ypT ypT0 ypT1 ypT2 ypT3 ypT4	67.3 67.8 66.2 37.2 32.6	<.001
Response @T-level* Complete response (ypT0) No complete response	67.3 48.6	<.001
Complete response Complete response No complete response	72.6 48.3	<.001
ypN ypN0 ypN1 ypN2	58.1 42.5 21.5	<.001
yp Nodal Status Negative Positive	58.1 35.0	<.001
RCT RT	51.7 45.7	.179
Adjuvant Chemotherapy Chemotherapy No Chemotherapy	48.5 52.6	.057

*4 nodal-positive patients.

**Table 3:** 10-year Overall Survival Univariate Analysis.

10-year Overall Survival Univariate Analysis Factor	10-Year Overall Survival (%)	p-Value
Age Split according to Median <65.8 ≥65.8	68.6 42.0	<.001
Sex Female Male	61.2 52.9	.032
cT cT2 cT3 cT4	67.1 58.1 31.9	<.001
cN cN0 cN1 cN2	60.7 57.1 46.7	.003
Clinical Nodal Status Negative Positive	60.7 54.0	.097
Downstaging @T-level Downstaging No Downstaging	66.8 45.9	<.001
Down-staging @N-level Downstaging No Downstaging	62.5 52.3	.001
Down-staging UICC-level Downstaging No Downstaging	65.6 46.7	<.001
ypT ypT0 ypT1 ypT2 ypT3 ypT4	66.8 69.8 71.1 45.1 36.7	<.001
Response @T-level* Complete response (ypT0) No complete response	66.8 54.7	.022
Complete response Complete response No complete response	72.1 54.4	.006
ypN ypN0 ypN1 ypN2	63.1 49.9 27.6	<.001
yp Nodal Status Negative Positive	63.1 41.7	<.001
Neoadjuvant Therapy RCT RT	57.7 49.3	.023
Adjuvant Chemotherapy Chemotherapy No Chemotherapy	55.6 56.0	.870

*4 nodal-positive patients.

Evaluation of the NAR scores categorized patients as low (NAR<8) in 14.4%, intermediate (NAR 8–16) in 50.8%, and high (NAR >16) in 33.1%. The NAR score could not be calculated in 15 patients (1.7%) due to missing values.

After performing a univariate Cox regression analysis, significant variables were used for the multivariate analysis to establish a risk prediction score for five-year DFS and five-year OS. Variables used for the multivariate five-year DFS absolute risk prediction model can be seen in **Table 4**; corresponding covariate means can be found in **Supplementary Table 1**. Evaluation of the model was performed by using the same data and reached an Area Under the Receiver Operating Characteristic (AUROC) of 0.722 and an overall good model quality with the lower bound of the 95% confidence interval of 0.684. The AUROC curve is visualized in **Supplementary Figure 1**. Variables used for the multivariate five-year OS absolute risk prediction model can be seen in **Table 5**; corresponding covariate means can be found in **Supplementary Table 2**. Evaluation of the model was performed by using the same data and reached an AUROC of 0.716 and an overall good model quality with the lower bound of the 95% confidence interval of 0.673. The AUROC curve is visualized in **Supplementary Figure 2**.

**Table 4:** Cox regression of variables in connection to 5-Year DFS Survival in the whole cohort.

Variables in the Equation								
	B	SE	Wald	df	Sig.	Exp(B)	95,0% CI for Exp(B)	
							Lower	Upper
Male Sex	.471	.136	12.012	1	.001	1.602	1.227	2.092
cT2			11.229	2	.004			
cT3	.354	.385	.845	1	.358	1.425	.670	3.030
cT4	.985	.424	5.380	1	.020	2.677	1.165	6.150
cN0			33.066	2	.000			
cN1	.402	.156	6.667	1	.010	1.494	1.102	2.027
cN2	1.112	.197	31.975	1	.000	3.040	2.068	4.468
No downstaging at the T-level	.837	.139	36.052	1	.000	2.309	1.757	3.035
No downstaging at the N-level	.967	.158	37.362	1	.000	2.631	1.929	3.588

The general formula for absolute risk prediction used was: $R(t)=1-{\left[Su_{0}(t)\right]}^{\text{exp}(\sum_{r=1\;}^{l}{\text{X}}_{r}{\text{β}}_{r}-\;\sum_{r=1\;}^{l}{\bar{\text{X}}}_{r}{\text{β}}_{r})}$ , where R is the risk of the event occurring in the calculated time period t (60 months in this case), l is the number of risk factors, Su_0_ the base survival probability at mean values, X_r_ the value of the corresponding risk factor, β_r_ the corresponding regression coefficient.

If used for a female example patient with cT3, cN1, no downstaging at the T-level, but downstaging at the N-level, the following result can be reached: R (60 months) = 1 – [0,670631]exp(((0,471368 x 0) + (0,353943 x 1) + (0,984558 x 0) + (0,401711 x 1) + (1,111706 x 0) + (0,836878 x 1) + (0,967378 x 0)) - ((0,471368 x 64.911783) + (0,353943 x 0.871338) + (0,984558 x 0.089172) + (0,401711 x 0.459873) + (1,111706 x 0.196178) + (0,836878 x 0.519745) + (0,967378 x 0.583439))) = 0.212121, which equals a 21.21% absolute risk for recurrence or death in the first five years after surgery. The DFS probability at the cohort’s mean values was 67.06%, and the base DFS probability at all covariates at zero was 95.27%.

**Table 5:** Cox regression of variables in connection to 5-Year Overall Survival in the whole cohort.

Variables in the Equation								
	B	SE	Wald	df	Sig.	Exp(B)	95,0% CI for Exp(B)	
							Lower	Upper
Age	.025	.007	11.399	1	.001	1.025	1.010	1.040
cT2			8.053	2	.018			
cT3	.573	.509	1.268	1	.260	1.774	.654	4.809
cT4	1.180	.553	4.547	1	.033	3.255	1.100	9.629
cN0			30.134	2	.000			
cN1	.425	.188	5.084	1	.024	1.529	1.057	2.212
cN2	1.305	.242	28.957	1	.000	3.687	2.292	5.931
No downstaging at the T-level	.663	.172	14.773	1	.000	1.941	1.384	2.721
No downstaging at the N-level	1.233	.205	36.292	1	.000	3.430	2.297	5.122
Short-Term RT	.267	.170	2.465	1	.116	1.305	.936	1.821

For the five-year OS absolute risk prediction model, the same general formula as for the DFS model was used. If used for a 65 year old example patient with cT3, cN1, downstaging at the T-level, but downstaging at the N-level and short-term RT, the following result can be reached: R (60 months) = 1 – [0.792959]^exp(((0.0247 x 65) + (0.573141 x 1) + (1.180085 x 0) + (0.424829 x 1) + (1.304917 x 0) + (0.663004 x 0) + (1.232566 x 1) + (0.266502 x 1)) - ((0.0247 x 64.911783) + (0.573141 x 0.871338) + (1.180085 x 0.089172) + (0.424829 x 0.459873) + (1.304917 x 0.196178) + (0.663004 x 0.519745) + (1.232566 x 0.583439) + (0.266502 x 0.236943)))^ = 0.272623, which equals a 27.26% absolute risk for death in the first five years after surgery.

The overall survival probability at the cohort’s mean values was 79.30%, and the base overall survival probability at all covariates at zero was 99.48%.

## Discussion

This retrospective rectal cancer registry was conducted to validate that downstaging in the N-level after neoadjuvant RCT had a more significant impact on DFS and OS than downstaging of the T-level, which was found in the previously published ABCSG-R02 trial (23). The ABCSG-R02 trial aimed to evaluate the efficacy expressed by downstaging at the T-level and safety of preoperative daily capecitabine plus weekly oxaliplatin in combination with RT in treating LARC (29). Furthermore, it was evaluated if tumor downstaging at the T-level and pCR acts as a surrogate for survival. When addressing these endpoints and analyzing their effects on survival rates, our study group was able to show that downstaging of the T-level does not influence OS but does influence DFS with a borderline significance. Assessment of the nodal status of these patients showed that downstaging in the N-level highly influences patient DFS and OS. However, a more significant number of patients was needed to confirm this finding clearly, which has now been performed in this retrospective analysis. The prognostic relevance of lymph node status in patient survival was demonstrated previously, regardless of the applied chemotherapeutic regimen (30–33). Patients with positive lymph nodes after neoadjuvant RCT in LARC had a higher risk for local recurrences and the development of distant metastases than patients with negative lymph nodes. Furthermore, our previous study demonstrates that the downstaging of the N-level acts as a better surrogate for survival than the downstaging of the T-level.

However, this retrospective analysis of 878 patients with LARC who were treated with neoadjuvant RCT or short-term RT followed by TME in five highly experienced centers in Austria could not confirm that only downstaging of the N-level and not of the T-level acts as a surrogate for survival. 10-year DFS and 10-year OS for patients with downstaging of the T-level or the N-level were significant better compared with patients with no downstaging. Clinical lymph node status before any treatment did not impact DFS or OS. The decisive factor for a good outcome is lymph-node negativity after neoadjuvant therapy.

In the univariate analysis, we found that the downstaging of the T-level and the downstaging of the N-level are surrogates for survival. Furthermore, based on multivariant analysis, we developed calculators for absolute risk prediction for 5-year DFS and 5-year OS. These prediction calculators are able to estimate the individual risk for recurrence or death within the first five years after surgery. While a dataset validation is ongoing, the prediction calculators offer an opportunity to incorporate the individual patient follow-up assessment to adjust follow-up in patients at high risk of recurrence. Furthermore, predictors can be used to select patients in clinical trials who would benefit from adjuvant chemotherapy based on their individual risk. Some parameters from the NAR score, such as cT and possible stage migration, were also significant factors in our predictive models. Nevertheless, our analysis showed that further factors like cN status, stage migration, short-course RT, age and gender are of importance for DFS and OS estimation. Compared to the TRG system, we included the lymph node status in our prediction model.

Improvement of OS is the most important goal when treating cancer patients and is the preferred primary clinical endpoint in studies. Its usefulness is limited by several disadvantages, requiring a higher number of patients, longer follow-up, and is associated with higher study costs. Therefore, identifying short-time surrogate endpoints for DFS or OS are useful for individualizing follow-up and adjuvant therapy. Surrogate endpoints in rectal cancer after neoadjuvant therapy are rare due to its complex validation and confirmation in phase III clinical trials. A pCR after neoadjuvant therapy and surgery is commonly associated with better outcomes compared to patients without pCR (14, 34). However, Petrelli et al. showed in a literature-based analysis of 22 randomized trials, that pCR and DFS are not surrogate endpoints for 5-year survival in rectal cancer (35). Nevertheless, we could demonstrate in our retrospective analysis, that patients with a pCR had a significantly better 10-year DFS and 10-year OS compared with patients without pCR. Furthermore, any response to therapy with T-downstaging or N-downstaging resulted in better survival compared to patients without response. This study was performed in a retrospective manner and thus might possess limited accuracy. Collected data on tumor stages did not include substages (e.g. cT3a, cT3b, etc.) and might therefore be a bias. Different time periods between treatment regimens and centers might be a confounding factor for this analysis, especially when comparing the RCT and RT cohorts.

Establishing a cancer registry is a complex process, especially when the data are collected retrospectively in different centers. The advantages of retrospective analysis include a large sample size, the participation of patients who are usually excluded from randomized clinical trials, the evaluation of a broad range of outcomes, and lower costs. Furthermore, the data are available quicker than in a prospective trial. Limitations of retrospective analysis include low internal validity, lack of quality control surrounding data collection, and susceptibility to multiple sources of bias for comparing outcomes. Six centers in Austria participated in our retrospective registry. Data were collected retrospectively utilizing existing data generated during routine clinical practice. Only a few centers had a locally established registry, but with different variables queried. The biggest challenge in our registry was merging the data from the centers across Austria. Strengths of our retrospective analysis are the high quality of local staging in all participating centers using standardized MRI and the large sample size. Nevertheless, of the discussed limitations, we could generate a prediction calculator for 5-year DFS and 5-year OS. The next step is to validate this prediction calculator in another cohort. This validation is still ongoing.

## Conclusion

Response to neoadjuvant RCT/RT regarding T-downstaging and N-downstaging in LARC resulted in better DFS and OS compared to patients without response. The most significant benefit was seen in patients with pCR. Furthermore, an easy to handle absolute risk prediction calculator for 5-year DFS and 5-year OS based on routinely collected clinical data was generated. The final dataset validation is ongoing.

## Data availability statement

The original contributions presented in the study are included in the article/**Supplementary Material**. Further inquiries can be directed to the corresponding author.

## Ethics statement

The studies involving humans were approved by Ethikkommission des Landes Oberösterreich. The studies were conducted in accordance with the local legislation and institutional requirements. The participants provided their written informed consent to participate in this study.

## Author contributions

GP: Conceptualization, Data curation, Formal analysis, Investigation, Methodology, Project administration, Supervision, Validation, Visualization, Writing – original draft, Writing – review & editing. FP: Conceptualization, Data curation, Formal analysis, Investigation, Methodology, Project administration, Supervision, Validation, Visualization, Writing – original draft, Writing – review & editing. JT: Data curation, Investigation, Supervision, Writing – original draft, Writing – review & editing. TB: Data curation, Investigation, Supervision, Writing – original draft, Writing – review & editing. HR: Data curation, Investigation, Supervision, Writing – original draft, Writing – review & editing. AD: Conceptualization, Data curation, Investigation, Supervision, Writing – original draft, Writing – review & editing. LW: Conceptualization, Data curation, Investigation, Methodology, Supervision, Writing – original draft, Writing – review & editing. RG: Data curation, Investigation, Supervision, Writing – original draft, Writing – review & editing. MG: Data curation, Investigation, Supervision, Writing – original draft, Writing – review & editing. DÖ: Conceptualization, Data curation, Formal analysis, Investigation, Methodology, Project administration, Supervision, Validation, Visualization, Writing – original draft, Writing – review & editing.

## Supplementary material

Supplementary Figure 1 AUROC curve of the 5-Year DFS prediction model with an AUROC of 0.722.

Supplementary Figure 2 AUROC curve of the 5-Year OS prediction model with an AUROC of 0.716.
