# Supplementary figures and images for: Prediction of survival after neoadjuvant therapy in locally advanced rectal cancer – a retrospective analysis
Source: Front Oncol. 2024 May 16;14:1374592. doi: 10.3389/fonc.2024.1374592 (PMC11137682; doi:10.3389/fonc.2024.1374592)

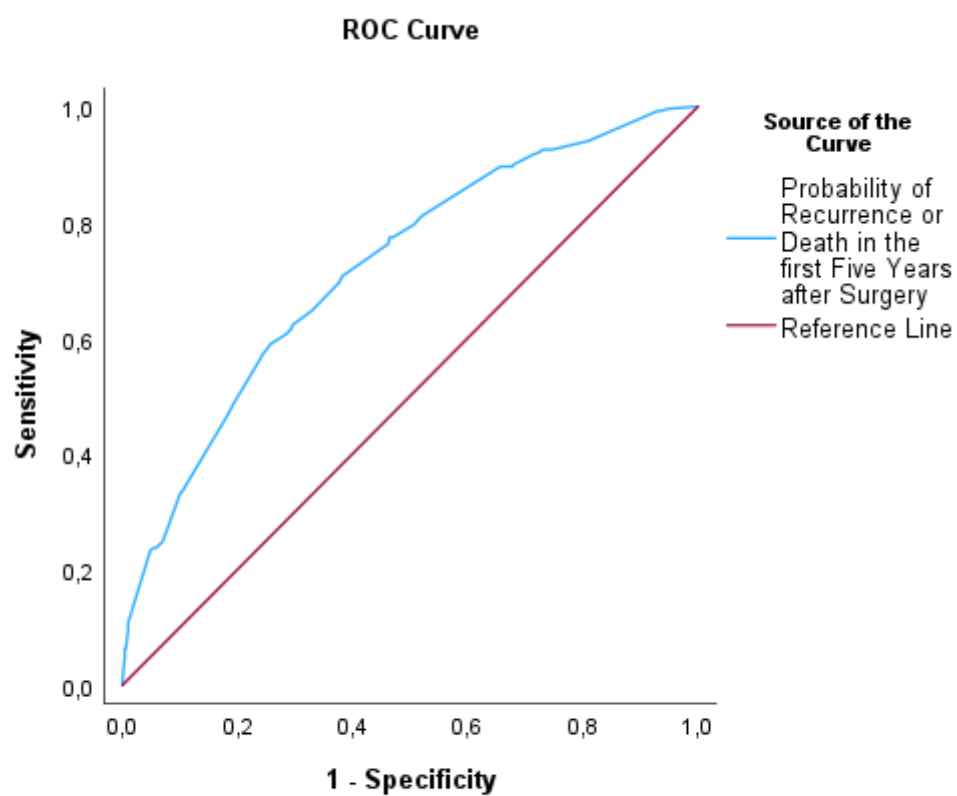

Supp. Figure 1. AUROC curve of the 5-Year DFS prediction model with an AUROC of 0.722.

Supplement: Supplementary Figure 1 — AUROC curve of the 5-Year DFS prediction model with an AUROC of 0.722. [file DataSheet_1.pdf]

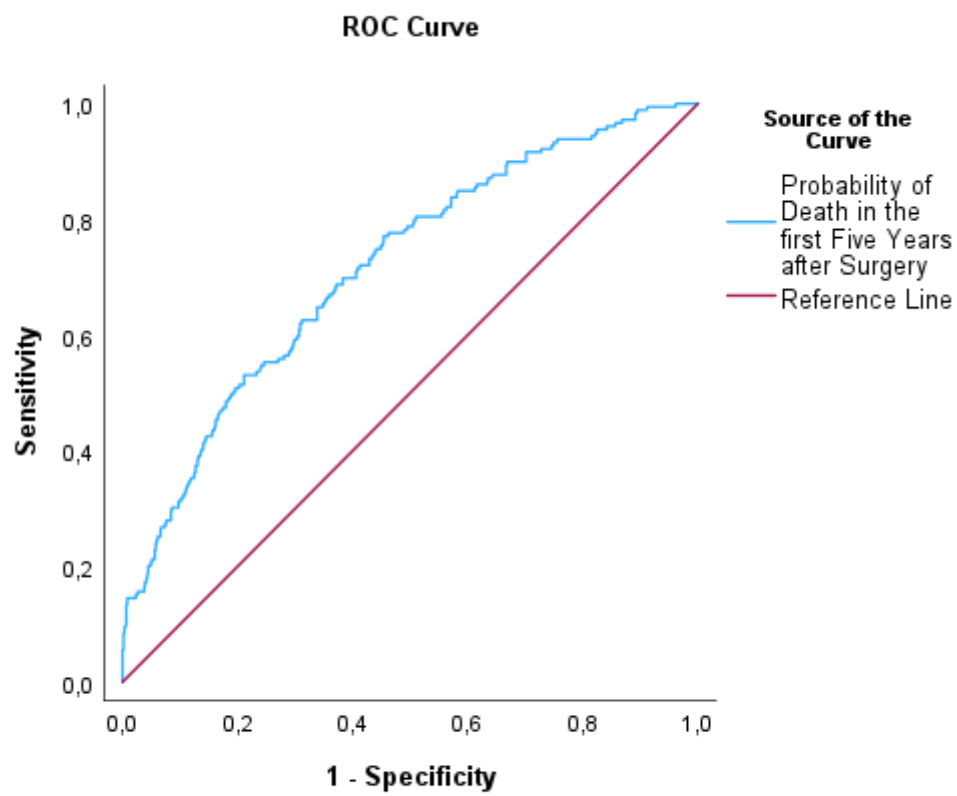

Supp. Figure 2. AUROC curve of the 5-Year OS prediction model with an AUROC of 0.716.

Supplement: Supplementary Figure 2 — AUROC curve of the 5-Year OS prediction model with an AUROC of 0.716. [file DataSheet_2.pdf]
