# Supplementary material for: Prediction of survival after neoadjuvant therapy in locally advanced rectal cancer – a retrospective analysis
Source: Front Oncol. 2024 May 16;14:1374592. doi: 10.3389/fonc.2024.1374592 (PMC11137682; doi:10.3389/fonc.2024.1374592)
Supplement: Supplementary file 3 [file Table_1.docx]

**Supplement Tables**

Supp. Table 1: five-year DFS corresponding covariate means

| Covariate | Mean |
| --- | --- |
| Male Sex | 0.658599 |
| cT3 | 0.871338 |
| cT4 | 0.089172 |
| cN1 | 0.459873 |
| cN2 | 0.196178 |
| No Downstaging at T-level | 0.519745 |
| No Downstaging at N-level | 0.583439 |

Supp. Table 2: five-year OS corresponding covariate means

| Covariate | Mean |
| --- | --- |
| Age | 64.911783 |
| cT3 | 0.871338 |
| cT4 | 0.089172 |
| cN1 | 0.459873 |
| cN2 | 0.196178 |
| No Downstaging at T-level | 0.519745 |
| No Downstaging at N-level | 0.583439 |
| Short-Term RT (instead of RCTx) | 0.236943 |
